# A Kinect-Based Real-Time Compressive Tracking Prototype System for Amphibious Spherical Robots

**DOI:** 10.3390/s150408232

**Published:** 2015-04-08

**Authors:** Shaowu Pan, Liwei Shi, Shuxiang Guo

**Affiliations:** 1 The Institute of Advanced Biomedical Engineering System, School of Life Science, Beijing Institute of Technology, No.5, Zhongguancun South Street, Haidian District, Beijing 100081, China; E-Mails: panshaowu.hit@gmail.com (S.P.); guoshuxiang@bit.edu.cn (S.G.); 2 Key Laboratory of Convergence Medical Engineering System and Healthcare Technology, the Ministry of Industry and Information Technology, Beijing Institute of Technology, No.5, Zhongguancun South Street, Haidian District, Beijing 100081, China; 3 Faculty of Engineering, Kagawa University, 2217-20 Hayashi-cho, Takamatsu, Kagawa 760-8521, Japan

**Keywords:** compressive tracking, Kinect, tracking system, real time, mobile robot

## Abstract

A visual tracking system is essential as a basis for visual servoing, autonomous navigation, path planning, robot-human interaction and other robotic functions. To execute various tasks in diverse and ever-changing environments, a mobile robot requires high levels of robustness, precision, environmental adaptability and real-time performance of the visual tracking system. In keeping with the application characteristics of our amphibious spherical robot, which was proposed for flexible and economical underwater exploration in 2012, an improved RGB-D visual tracking algorithm is proposed and implemented. Given the limited power source and computational capabilities of mobile robots, compressive tracking (CT), which is the effective and efficient algorithm that was proposed in 2012, was selected as the basis of the proposed algorithm to process colour images. A Kalman filter with a second-order motion model was implemented to predict the state of the target and select candidate patches or samples for the CT tracker. In addition, a variance ratio features shift (VR-V) tracker with a Kalman estimation mechanism was used to process depth images. Using a feedback strategy, the depth tracking results were used to assist the CT tracker in updating classifier parameters at an adaptive rate. In this way, most of the deficiencies of CT, including drift and poor robustness to occlusion and high-speed target motion, were partly solved. To evaluate the proposed algorithm, a Microsoft Kinect sensor, which combines colour and infrared depth cameras, was adopted for use in a prototype of the robotic tracking system. The experimental results with various image sequences demonstrated the effectiveness, robustness and real-time performance of the tracking system.

## 1. Introduction

To execute various tasks autonomously in ever-changing environments, it is critical for a robot to be able to detect its surroundings. As a kind of feasible sensor with advantages of low cost, low power consumption and strong adaptability [1], digital cameras have been widely used in robotics to guide electromechanical devices and realise intelligent methods. In a machine vision-based robot, the visual tracking system plays a very important role in realising diverse robotic functions, such as autonomous navigation [2,3], path planning [4,5], visual servoing [6,7], robot-human interaction [8,9], *etc.*

In general, existing tracking algorithms can be categorised as estimation-based and classification-based algorithms [10]. Estimation-based or generative algorithms model the target based on appearance features and then search for it in each frame of the visual stream [11]. Examples of this type of algorithm, which are mainly driven by innovations in appearance models, include MeanShift [12], particle filter-based algorithms [13], Incremental Visual Tracking (IVT) [14] and optical flow-based algorithms [15]. Classification-based or discriminative algorithms treat tracking as a binary pattern recognition problem and try to separate the target from the background [16]. This type is usually built upon pattern recognition algorithms, such as support vector machine (SVM) [17], Bayes classifier [18], K-means [19], *etc.* Most studies on visual tracking are performed on high-performance computers and are evaluated with standard benchmark sequences, each of which contain controlled disturbances in the target or environment.

Compared with studies of tracking algorithms based on commercial computers in a controlled laboratory environment, designing a reliable visual tracking system for mobile robots is an even more challenging task for two reasons [20,21]. First, the appearance of targets varies due to pose or scale changes, random motion and occlusion, making it difficult to establish a reliable appearance model [22]. Second, the ambient environment of robots can be easily disturbed by variations in illumination, camera vibration and outside interference, which may cause problems of drift or target loss [23]. More importantly, most state-of-the-art studies aimed at improving tracking precision, adaptability and robustness, overlooking the real-time performance and computational requirements of algorithms. This has led to a situation in which most existing algorithms are unsuitable for application to mobile robots equipped with embedded microprocessors and limited power sources.

To realise flexible and economical underwater exploration, in 2012 our team designed an amphibious spherical robot inspired by turtles [24,25,26]; an improved version conceptualised as “all programmable” was designed in 2014 [27]. Given the special working environments and application functions of the amphibious robot, an improved RGB-D visual tracking algorithm with dual trackers is proposed and implemented in this paper. Compressive tracking (CT) was selected as the basis of the proposed algorithm to process colour images from a RGB-D camera, and a Kalman filter with a second-order motion model was added to the CT tracker to predict the state of the target, select candidate patches or samples and reinforce the tracker’s robustness to high-speed moving targets. In addition, a variance ratio features shift (VR-V) tracker with a Kalman prediction mechanism was adopted to process depth images from a RGB-D camera. A visible and infrared fusion mechanism or feedback strategy is introduced in the proposed algorithm to enhance its adaptability and robustness. Through the feedback strategy, the depth tracking results were used to assist the CT tracker in updating classifier parameters at an adaptive rate. To evaluate the effectiveness of the algorithm, a Microsoft Kinect device, which is a combination of colour and depth cameras, was adopted for use in a prototype of the robotic tracking system. Experimental results in various scenarios demonstrated the effectiveness, robustness and real-time performance of the tracking system. Furthermore, drift, which is an obvious problem of CT, was partly solved in the proposed algorithm.

The rest of this paper is organised as follows: an overview of related work, which covers our amphibious spherical robot and CT, is presented in Section 2. Three major areas of the proposed algorithm are elaborated in Section 3 and the structure of the prototype tracking system for our amphibious spherical robot and experimental results on various benchmark sequences are covered in Section 4. Section 5 presents the conclusion and suggestions for relevant follow-up research.

## 2. Related Works and Application Requirements

### 2.1. Amphibious Spherical Robot

**Figure 1:**
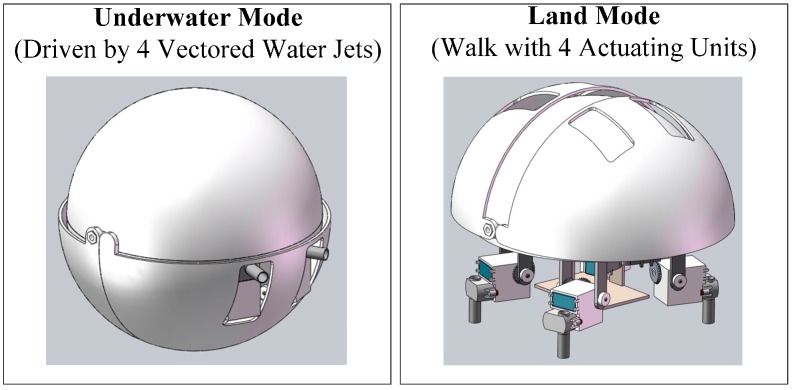
Diagram of our spherical amphibious robot.

As introduced in references [24] and [27], an amphibious spherical robot was proposed for covert missions or tasks in narrow spaces that a typical autonomous underwater vehicle (AUV) could not complete. As shown in Figure 1, the shell of the robot consisted of a hemispheric upper hull (250 mm in diameter) and two quarter-sphere lower hulls (266 mm in diameter) that can open and close. The hard upper hull was waterproof and served to protect the internal electronic devices and batteries from collisions.

**Figure 2:**
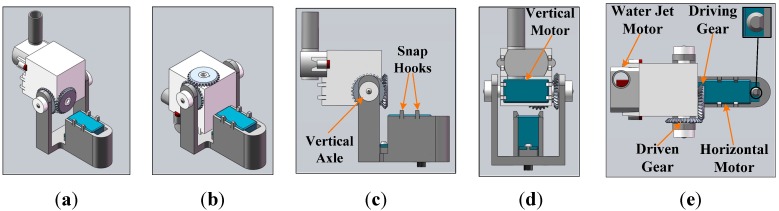
Diagram of actuating units and related driving parts. (**a**) Stretching mode; (**b**) Huddling mode; (**c**) Frontal view; (**d**) Profile view; and (**d**) Top view.

As shown in Figure 2, four actuating units, each consisting of two servomotors and a water jet motor, were symmetrically installed under the upper hull. When the robot was in underwater mode, the lower hulls closed, and four water jet motors in the actuating units provided vectored thrust though strip-type holes in the lower hulls to realise motion with six degrees of freedom (DOF). When the robot was in land mode, the lower hulls opened, and the actuating units stretched out to walk quadrupedally under the driving force of eight servomotors. To facilitate upgrades, three-dimensional (3-D) printing technology, a minimal Xilinx Zynq-7000 SoC system and an embedded computer equipped with an Intel Atom processor were adopted to fabricate the robot [26].

Due to the unique mechanical structure and the specialised potential application scenarios, customising the tracking system of the amphibious spherical robot was a challenging task. First, the upper hull of the robot, in which electronic devices, scientific instruments and batteries were installed, provided only a very narrow enclosed space. Hence, high-speed computers, which consume considerable power and generate a great deal of heat, could not be adopted in this mobile robot and consequently, the real-time performance of the adopted tracking algorithm became a critical problem. Second, as the robot may work under diverse land and underwater conditions, robustness to environmental disturbances caused by camera vibration and occlusion was essential to the adopted tracking algorithm. Third, the field of view of the camera was very narrow due to limitations imposed by the installation space, making it crucial to improve the adaptability of the tracking algorithm to small and high-speed moving targets [28]. Moreover, the visual tracking system of the robot was intended for use chiefly in autonomous navigation and in guiding electromechanical devices. Consequently, its tracking precision and effectiveness should be acceptable.

### 2.2. Real-Time Compressive Tracking Algorithms

**Figure 3:**
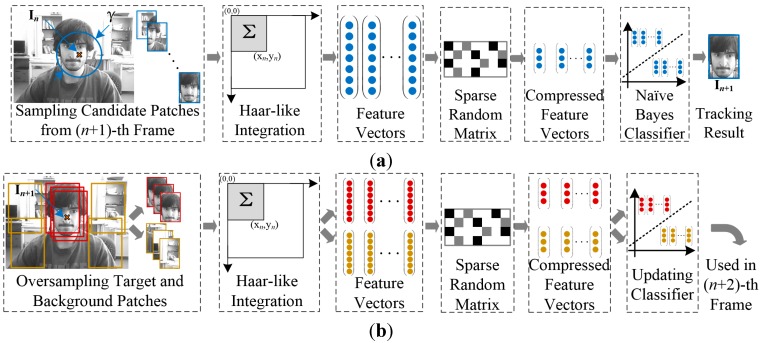
Diagram of the original CT algorithm. (**a**) Tracking at the (*n* + 1)-th frame; and (**b**) Updating classifier after Tracking at the (*n* + 1)-th frame.

In 2012, Zhang *et al.* proposed the CT algorithm, which provided a novel framework for developing real-time tracking algorithms for mobile robots [29]. Figure 3 shows the main components of the CT algorithm. As an effective and efficient discriminative algorithm, CT consists of two stages: Tracking and updating. In the tracking stage, candidate image patches or samples of the target of the (*n* + 1)-th frame are sampled around I_*n*_, which is the tracking result at the *n*-th frame. Then, integral vectors of these patches are calculated by accumulation. Based on compressive sensing theory, the high-dimensional integral vectors of samples are compressed to extract Haar-like features using a static measurement matrix. The process of compression or measurement can be denoted as v=Ru, where $u\in {\mathbb{R}}^{n}$ indicates the integral vectors and $v\in {\mathbb{R}}^{m}$ indicates the compressed feature vectors with dimensions m≪n. R is a very sparse random matrix, the entries of which were defined as:

(1) $$r_{i,j}=\sqrt{\text{s}}\times \{\begin{array}{c} \text{+1,with probability }\frac{1}{\text{2s}}\\ \text{0,with probability 1}-\frac{1}{\text{s}}\\ -\text{1,with probability }\frac{1}{\text{2s}} \end{array}$$

where s is set to *m*/4. For each row of R, fewer than four entries are non-zero, which results in very low computational complexity of the compression process. Then, the low-dimensional feature vectors are entered into an online learning Naïve Bayes classifier. The sample with maximal classifier response is set to the target for determining ${\text{I}}_{n\text{+1}}$. In the updating stage, training samples of the target and the background are oversampled according to the tracking result at the *(n* + 1)-th frame (${\text{I}}_{n\text{+1}}$), and the compressed feature vectors of the training samples are used to update the parameters of the Naïve Bayes classifier, which will be used in the tracking stage of the (*n* + 2)-th frame.

The CT algorithm succeeded in real-time performance and provided a low-cost and lossless way to compress image information and exact appearance features by using compressive sensing theory. However, CT samples the candidate patches within a predefined radius around the tracking result of the previous frame. Consequently, tracking precision relies heavily on the value of the radius, and CT is unable to track targets in complex or high-speed motion. Moreover, as the parameters of the classifier are updated at a constant rate, the tracking results continue to drift once a deviation occurs. Furthermore, the target will be lost if it is fully occluded for a long time. In addition, scale invariance is not taken into account in CT, which exacerbates the problem of tracking drift.

To compensate for the deficiencies of CT, Xu *et al.* proposed an improved CT algorithm containing two weighted classifiers that processed previous and current samples separately [30]. Chen *et al.* proposed a scalable CT algorithm that used optical flow to estimate the size of the target [18]. Yan *et al.* combined CT with sparse representation and proposed a kernel sparse tracking algorithm [31]. Zhang *et al.* added a coarse-to-fine search strategy to CT and proposed a fast compressive tracking (FCT) algorithm [32]. However, improvements introduced in the above studies added significant complexity to the algorithm or only solved a specialised problem, limiting their applications in our amphibious spherical robot.

## 3. Proposed Algorithm

On the other hand, the drift problem of CT is associated with the accumulation of tracking errors caused by the online self-updating process of the classifier. Therefore, adding a semi-supervised or feedback mechanism to the updating process should be an efficient method for addressing the drift problem [10].

On the other hand, as digital images are projections of 3-D objects on a two-dimensional (2-D) plane, visual tracking is a process of estimation based on incomplete information. Consequently, stereovision and multi-sensor fusion, which provide additional information about the target (depth, temperature, velocity, *etc.*), are effective means of improving the precision and robustness of tracking algorithms. For the reason that a depth image directly provides the three-dimensional information of the surface of an object, it can be used in RGB-D tracking algorithms to handle thorny problems such as occlusion, drift and clutter environment. The Microsoft Kinect, which is a low-cost commercial RGB-D camera proposed in 2012, has promoted the development of RGB-D tracking algorithms in recent years [33]. As Kinect is close to a medium-resolution stereo camera in resolution and precision of the depth measurement [34], it has been successfully used to fabricate robust tracking systems by combining colour and depth features [35]. Because most available vision algorithms designed for colour images cannot be directly extended to detect or track targets in depth images, histograms of oriented gradients (HOG), histogram of oriented normal vectors (HONV) and local ternary patterns (LTP) were usually adopted to extract depth features of targets in existing Kinect-based RGB-D tracking algorithms [36,37]. However, these algorithms are unsuitable to embedded microprocessor-based robotic applications in consideration of their real-time performance [38,39].

**Figure 4:**
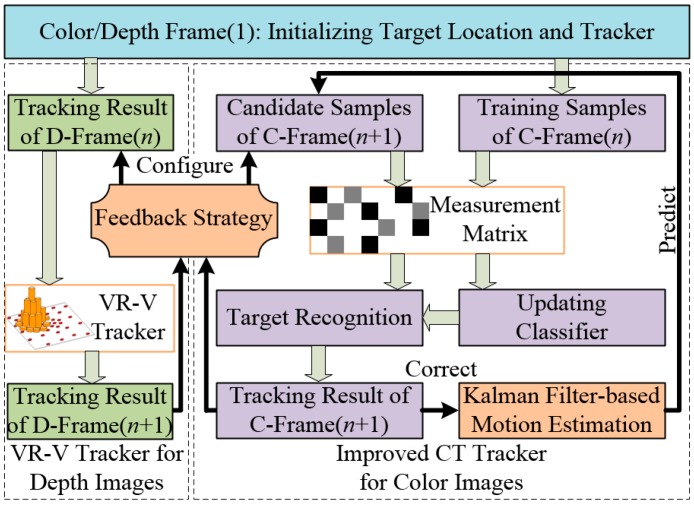
Main components of the proposed algorithm.

Making use of the features of Kinect and CT, an improved visual tracking algorithm for application of our amphibious spherical robot is presented based on the concept of multi-feature fusion and feedback presented in this paper. The main components of the proposed algorithm are shown in Figure 4. Microsoft Kinect was adopted as a compound RGB-D image sensor for the prototype tracking system, and dual trackers were implemented to process colour and depth images separately. An improved CT tracker with a Kalman prediction mechanism was deployed as the main tracker to process colour images (C-Frame), and a VR-V tracker was implemented as an assist tracker to process depth images (D-Frame). To realise sensor fusion and improve robustness of the proposed algorithm, a feedback strategy inspired by expert control was designed to fuse the tracking results of the trackers. The major contributions of the proposed algorithm are in three areas: Motion estimation for CT, VR-V-based depth tracking and the feedback strategy.

### 3.1. Visual Compressive Tracking with Motion Estimation

**Figure 5:**
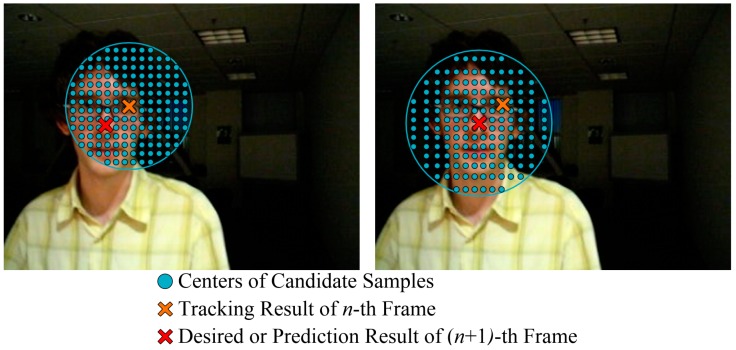
Diagram of candidate patch sampling.

As mentioned in Section 2, the original CT algorithm samples candidate patches around the previous tracking result in a static radius set manually; this process can be denoted as:

(2) $$D^{\gamma }=\left\{z|\left\|I\text{(}z{\text{)-I}}_{n}\right\|\text{<}\gamma \right\}$$

where $D^{\gamma }$ is a set of candidate samples at the (*n* + 1)-th frame, ${\text{I}}_{n}$ is the centre of the target at the *n*-th frame, and γ is the sampling radius, which was set to 25 in the sample CT program [29]. This sampling method reduces the robustness of CT to targets in complex or high-speed motion. As shown in the left-hand image of Figure 5, because the radius is static, the desired tracking result ${\tilde{I}}_{n+1}$ may be on the edge of or even outside the sampling region. This can lead to visual drift or to missing the target entirely. This problem can be partially solved by setting the sampling radius to a larger value, but this will significantly increase the number of candidate patches to be sampled and processed, which decreases the superiority of CT in real-time performance.

As shown in the right-hand image of Figure 5, to balance the real-time performance and robustness of CT, the centre of the sampling region was modified so that it was located at the predicted tracking result for the (*n* + 1)-th frame, and a Kalman filter was deployed to estimate the sampling centre. Considering the prediction and tracking errors of the improved CT tracker, the location and motion tendency of the target can be estimated with the linear dynamic model denoted as:

(3) $$X_{n+1}=\Phi X_{n}+\beta W_{n}$$

(4) $$Y_{n+1}=HX_{n+1}+\alpha V_{n}$$

(5) $$\Phi =\left(\begin{array}{cccc} 1 & 0 & \Delta t & 0\\ 0 & 1 & 0 & \Delta t\\ 0 & 0 & 1 & 0\\ 0 & 0 & 0 & 1 \end{array}\right)$$

(6) $$H=\left(\begin{array}{c} \begin{array}{cccc} 1 & 0 & 0 & 0 \end{array}\\ \begin{array}{cccc} 0 & 1 & 0 & 0 \end{array} \end{array}\right)$$

(7) $$X_{n}={\left(x_{n},y_{n},x_{n}-x_{n-1},y_{n}-y_{n-1}\right)}^{\text{T}}$$

(8) $$Y_{n}={\left({\tilde{x}}_{n},{\tilde{y}}_{n}\right)}^{\text{T}}$$

where Φ is the transfer matrix, H is the measurement matrix, α and β are adjustable parameters of the filter, $W_{n}$ is the noise vector of prediction and $V_{n}$ is the noise vector of measurement. The real state vector $X_{n}$ contains 2-D position and velocity information on the target, and the measured state vector $Y_{n}$ contains 2-D position information on the target. As most objects in smooth motion can be described by a third-order motion model and error in the tracking process is common, a second-order motion model was adopted in this filter to increase reliability.

The prediction process can be described as:

(9) $${\hat{X}}_{n+1|n}=\Phi {\hat{X}}_{n|n}$$

(10) $$P_{n+1|n}=\Phi P_{n|n}\Phi^{\text{T}}+\beta Q_{n+1}\;$$

where **P**_*n*+1|*n*_ is the covariance matrix of noise. After predicting the motion state at the (*n* + 1)-th frame, the sampling centre was set to ${\hat{I}}_{n+1}$, which was extracted from the prediction vector ${\hat{X}}_{n+1|n}$, and the candidate samples centred at ${\hat{I}}_{n+1}$ were collected based on a 2-D Gaussian distribution. As shown in the right-hand image of Figure 5, the density of candidate samples decreased with distance from the predicted location, which was closer to the actual position of the target. Consequently, as the number of samples would not increase exponentially with the sampling radius, it was possible to track high-speed moving targets using larger search regions in real-time. In addition, a matrix defining the Gaussian location of samples was calculated in advance to accelerate the sampling process.

After the CT tracking stage, which processed candidate samples and output the tracking result, the correction process can be described as:

(11) $$K_{n+1}=P_{n+1|n}H^{\text{T}}{\text{(}HP_{n+1|n}H^{\text{T}}+\alpha R_{n+1}\text{)}}^{-1}$$

(12) $${\hat{X}}_{n+1|n+1}={\hat{X}}_{n+1|n}+K_{n+1}\text{(}Y_{n+1}\text{-}H{\hat{X}}_{n+1|n}\text{)}$$

(13) $$P_{n+1|n+1}=\text{(}I\text{ -}K_{n+1}H\text{)}P_{n+1|n}$$

where $K_{n+1}$ is the Kalman gain matrix at the (*n* + 1)-th frame, I is a unit matrix, ${\hat{X}}_{n+1|n}$ is the measured state vector containing the tracking result and ${\hat{X}}_{n+1|n+1}$ is the filtered state vector. In this way, the correction process corrected parameters of the filter with residual error and the tracking result at the (*n* + 1)-th frame. Visual tracking is an inaccurate estimation process the results of which often incorporate error and drift caused by ever-changing environments and targets. Therefore, the coefficient vectors (α and β) of the noise matrix were adjusted adaptively by the feedback strategy, which will be introduced in Section 3.3.

### 3.2. Depth Tracking Based on Improved VR-V

As mentioned in Section 2, the original CT algorithm updates parameters of the Naïve Bayes classifier in the tracking process for each frame. As a result, if the target were fully occluded for a long time, the classifier would learn the features of the occluding object rather than the target, and the target would ultimately be lost.

Because the motion of a target is continuous, it is clear that the distance between the camera and targets would not change markedly in most of the time when there is no occlusion occurring. Similarly, the depth value of the target region would decrease significantly when occlusion occurs. Consequently, the occlusion problem of tracking algorithms can be alleviated or even handled by utilising depth images captured by RGB-D cameras. Besides, the adaptability of the entire tracking system was improved by a fusion of colour and depth image features, as the depth features of targets have some robustness to environmental disturbance.

**Figure 6:**
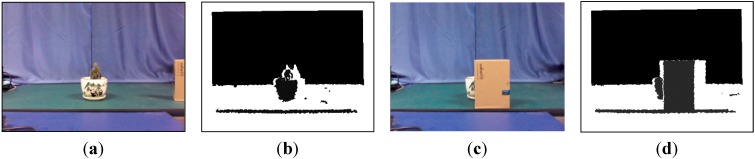
Colour and depth images captured by the Kinect. (**a**) Colour image; (**b**) Depth image; (**c**) Colour image after occlusion; and (**d**) Depth image after occlusion.

As shown in Figure 4, a VR-V tracker was deployed to assist the CT tracker. As an updated version MeanShift, VR-V selects the best discriminative features of targets online and then locates targets with multiple MeanShift trackers [40]. As shown in Figure 6b,d, the grey histogram of the target region in a depth image describe the contour and surface features of the target roughly, so MeanShift-based VR-V algorithm are able to track targets in depth images. And the selection of tracking features provides an adaptive online learning mechanism to track target, the depth value of which is changing over time. Furthermore, the real-time performance of the entire tracking system can be ensured because VR-V is a concise algorithm with high efficiency.

As shown in Figure 6a,c, the improved CT tracker cannot be aware that the target in colour images is partly or fully occluded, but the histogram-based VR-V tracker for depth images is able to detect the exception and instruct the CT tracker to temporarily stop the classifier update. To utilise this indication, a VR-V tracker with a Kalman prediction mechanism was adopted to process depth images. The prediction and correction processes of the Kalman filter were similar to the improved CT algorithm, and the depth tracking position provided by the VR-V tracker could be used as reference information for the improved CT tracker, which will be introduced in Section 3.2.

**Figure 7:**
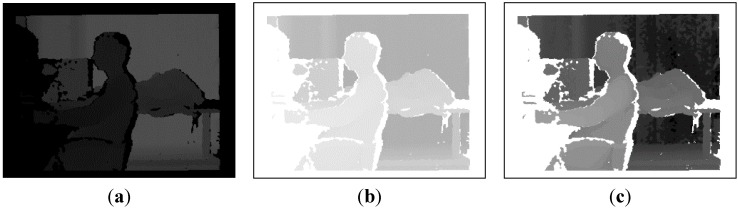
Diagram of depth image enhancement. (**a**) Before enhancement; (**b**) After invert; and (**c**) After equalization.

In actual experiments, the grey pixel value of depth images ranged from 0 to 255, but the effective working distance of the Kinect was only 1.0–1.5 m, which made the image mostly dark, with most of the pixels concentrated in the 200–255 range, as shown in the left image of Figure 7. To improve the imaging quality and thereby emphasise features of the target, an enhancement process was designed for use after image acquisition. As shown in the middle and right-hand images of Figure 7, the values of pixels were flipped, and a histogram equalisation operation was performed. As the depth values of smooth surfaces and remote objects may be inaccurate, the VR-V tracker may fail in tracking if the histogram of the target in depth images is unstable. To cope with this situation, the VR-V tracker will be reset when the tracking error is too large, which will be introduced in Section 3.3.

### 3.3. Feedback Strategy for Adaptive Online Learning

As mentioned in Section 2.2, a fatal defect of CT is that the parameters of the classifier are updated at a constant rate, which leads to tracking failure when the target is fully occluded. A dual tracker framework was designed to improve CT algorithm in Section 3.1 and Section 3.2. And a feedback strategy inspired by expert control was introduced in this section to fuse tracking results of both trackers and realise the adaptive updating of the parameters of the CT tracker.

Table 1 shows the status transition of the feedback strategy. The strategy tried to evaluate working status of the tracking system by analysing the quality of tracking results, detect occlusion by check histogram similarity of depth images, and improve robustness of the proposed tracking algorithm with an adaptive parameter updating mechanism. In Table 1, I_C(*n*)_ (I_D(*n*)_) represents the tracking results at the *n*-th colour (depth) frame, *s*_hist_ represents the histogram similarity measured with Bhattacharyya coefficient between *n*-th and (*n* + 1)-th depth frame, γ represents the sampling radius of the CT tracker, $\alpha_{\text{CT}}\text{,}\beta_{\text{CT}}$ represents the parameters of the Kalman filter adopted in the CT tracker and ${\text{λ}}_{0}{\text{,ε}}_{0}{\text{,δ}}_{0}$ are boundary constants that control the adjustment of parameters.

**Table 1:** Status transition table of the feedback strategy.

No.	I_C(*n* + 1)_ − I_D(*n* + 1)_	I_D(*n* + 1)_ − I_D(*n*)_	*s*_hist_	Adjust Parameter	Classifier Update
1	$\left|{\text{I}}_{\text{C(}n\text{+1)}}-{\text{I}}_{\text{D(}n\text{+1)}}\right|{\text{<λ}}_{0}$	$\left|{\text{I}}_{\text{D(}n\text{+1)}}-{\text{I}}_{\text{D(}n\text{)}}\right|{\text{<ε}}_{0}$	$s_{\text{hist}}{\text{<δ}}_{0}$	$\gamma =\gamma_{0}$	True
2	${\text{λ}}_{0}\text{<}\left|{\text{I}}_{\text{C(}n\text{+1)}}-{\text{I}}_{\text{D(}n\text{+1)}}\right|{\text{<λ}}_{1}$	$\left|{\text{I}}_{\text{D(}n\text{+1)}}-{\text{I}}_{\text{D(}n\text{)}}\right|{\text{<ε}}_{0}$	$s_{\text{hist}}{\text{<δ}}_{0}$	$\alpha_{\text{CT}}$,$\beta_{\text{CT}}$,$\gamma =1.2\gamma_{0}$	True
3	$\left|{\text{I}}_{\text{C(}n\text{+1)}}-{\text{I}}_{\text{D(}n\text{+1)}}\right|{\text{>λ}}_{1}$	$\left|{\text{I}}_{\text{D(}n\text{+1)}}-{\text{I}}_{\text{D(}n\text{)}}\right|{\text{<ε}}_{0}$	$s_{\text{hist}}{\text{<δ}}_{0}$	$\gamma =1.5\gamma_{0}$	False
4	$\left|{\text{I}}_{\text{C(}n\text{+1)}}-{\text{I}}_{\text{D(}n\text{+1)}}\right|{\text{<λ}}_{0}$	$\left|{\text{I}}_{\text{D(}n\text{+1)}}-{\text{I}}_{\text{D(}n\text{)}}\right|{\text{>ε}}_{0}$	$s_{\text{hist}}{\text{<δ}}_{0}$	$\gamma =1.2\gamma_{0}$, Reset VR-V	True
5	${\text{λ}}_{0}\text{<}\left|{\text{I}}_{\text{C(}n\text{+1)}}-{\text{I}}_{\text{D(}n\text{+1)}}\right|{\text{<λ}}_{1}$	$\left|{\text{I}}_{\text{D(}n\text{+1)}}-{\text{I}}_{\text{D(}n\text{)}}\right|{\text{>ε}}_{0}$	$s_{\text{hist}}{\text{<δ}}_{0}$	$\alpha_{\text{CT}}$,$\beta_{\text{CT}}$, $\gamma =1.5\gamma_{0}$ Reset VR-V	True
6	$\left|{\text{I}}_{\text{C(}n\text{+1)}}-{\text{I}}_{\text{D(}n\text{+1)}}\right|{\text{>λ}}_{1}$	$\left|{\text{I}}_{\text{D(}n\text{+1)}}-{\text{I}}_{\text{D(}n\text{)}}\right|{\text{>ε}}_{0}$	$s_{\text{hist}}{\text{<δ}}_{0}$	Reinitialization	False
7	$\left|{\text{I}}_{\text{C(}n\text{+1)}}-{\text{I}}_{\text{D(}n\text{+1)}}\right|{\text{<λ}}_{0}$	$\left|{\text{I}}_{\text{D(}n\text{+1)}}-{\text{I}}_{\text{D(}n\text{)}}\right|{\text{<ε}}_{0}$	${\text{δ}}_{0}\text{<}s_{\text{hist}}{\text{<δ}}_{1}$	$\gamma =1.2\gamma_{0}$	True
8	$\left|{\text{I}}_{\text{C(}n\text{+1)}}-{\text{I}}_{\text{D(}n\text{+1)}}\right|{\text{<λ}}_{0}$	$\left|{\text{I}}_{\text{D(}n\text{+1)}}-{\text{I}}_{\text{D(}n\text{)}}\right|{\text{<ε}}_{0}$	$s_{\text{hist}}{\text{>δ}}_{1}$	$\gamma =1.5\gamma_{0}$	False
9	$\left|{\text{I}}_{\text{C(}n\text{+1)}}-{\text{I}}_{\text{D(}n\text{+1)}}\right|{\text{>λ}}_{1}$	$\left|{\text{I}}_{\text{D(}n\text{+1)}}-{\text{I}}_{\text{D(}n\text{)}}\right|{\text{>ε}}_{0}$	$s_{\text{hist}}{\text{>δ}}_{1}$	Reinitialization	False

Under condition 1, if the tracking results of both trackers are almost consistent, indicating that there is little interference factors affecting the tracking system, the CT tracker will update the classifier parameters at a constant rate. Under conditions 2 and 7, if the tracking results of the CT tracker are not so consistent or the depth histogram of the target changes in certain limits, indicating that the target may move at a fast speed or the scale of the target may be varying, the search region of the candidate patches will be enlarged and parameters of the Kalman filter will be selectively adjusted. Under conditions 3 and 8, if the tracking results of the CT tracker are inconsistent or the depth histogram of the target changes significantly, indicating a high likelihood that the environmental conditions or target model has deteriorated (partly occlusion, illumination variance or pose change), the CT tracker will stop updating and try to search the target in a much larger region. Under conditions 4 and 5, if the tracking results of the CT tracker are consistent while the tracking results of the VR-V tracker are inconsistent, which may be caused by complex motion of the target or instability of the infrared tracking subsystem, the parameters of the Kalman filter will be adjusted, the search region of the CT tracker will be enlarged and the target position of VR-V tracker will be reset to I_C(*n* + 1)_. Under conditions 6 and 9, if the tracking results of the two trackers differ considerably, the target may have left the field of view or may have been lost. Therefore, classifier updating will terminate for 10 frames and then attempt to reinitialise the valid tracking process after recovery of the target. In these cases, the feedback extends the stable margin of the CT tracker by limiting the self-updating, enlarging the search region and adaptively adjust parameters.

## 4. Experiment and Discussion

### 4.1. Experimental Environment and Tracking System Prototype

**Figure 8:**
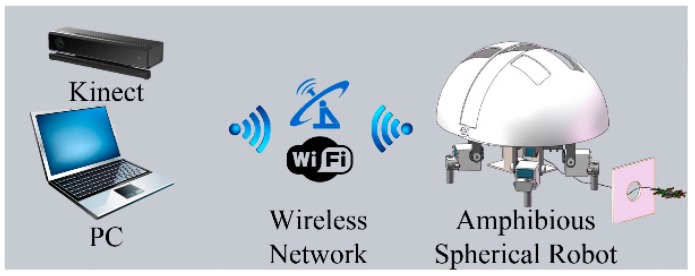
Prototype of the robotic tracking system.

As the study was performed to design a real-time, robust and effective algorithm for our amphibious spherical robot, both robot-based and computer-based experiments were designed to test its performance in virtual and actual scenarios with occlusion, irregular motion, illumination variation and other forms of interference. Experimental tests on the proposed algorithm consisted of three parts:

(1) In the benchmark test, the improved CT algorithm, which contained a motion estimation mechanism and ran on a computer (Intel Core i7-4712MQ, 8 GB RAM), was tested on seven standard benchmark sequences compared with the original CT algorithm and the online multiple-instance learning (MIL) algorithm [41].
(2) In the actual test, the proposed algorithm, running on the same computer, was deployed to process colour and depth image sequences captured by a Kinect compared with MIL algorithm, RGBD baseline algorithm and RGBDOcc algorithm [38].
(3) In the robotic test, the proposed algorithm was implemented to guide the motion of our amphibious spherical robot. As shown in Figure 8, due to limitations imposed by the size of the current version of our robot, a preliminary prototype of the robotic tracking system for the algorithm evaluation was fabricated, in which a Kinect was installed in the region of activity of the robot, where it monitored the motion of the robot while the proposed algorithm, running on the same computer, tracked a specialised guiding object and controlled the robot via the wireless local network as it followed the object.

### 4.2. Experimental Results

In the benchmark and actual tests, two metrics were used to evaluate the improved CT and proposed algorithm with CT and MIL. The first metric was the success rate (SR) of the benchmark sequences. The success rate of a frame was defined as:

(14) $$score=\frac{area(ROI_{T}\cap ROI_{G})}{area(ROI_{T}\cup ROI_{G})}$$

where *ROI*_T_ is the tracked bounding box, *ROI*_G_ is the ground truth bounding box and area(⋅) denotes the number of pixels in the region. If the score is larger than the given threshold (0.5 in this paper) in a frame, it is counted as a success [32]. The second metric is the centre location error (CLE), which is the Euclidean distance between the central points of the tracked bounding box and the ground truth bounding box. Given that a tracker may be sensitive to the initial conditions or parameters of the target in the first frame, various test results of the same algorithm can be obtained with different initial bounding boxes of the target. For a fair performance evaluation, the initial position and scale size of the target were specified according to the data of ground truth bounding boxes provided by [38]. Therefore, although there was no modification of the CT and MIL programs, the experimental results in this paper may be slightly poorer than the counterparts reported previously [32,41].

Table 2 lists the major challenging factors of the test image sequences for the benchmark and actual tests. The sequences “Coke”, “Couple”, “Liquid”, “Suv”, “Tiger1”, “Tiger2” and “Fish”, taken from [42], were used to test the improved CT algorithm, which was a single combination of CT and motion estimation based on the Kalman filter (elaborated in Section 3.1). The sequences “Note”, “Book”, “Glove1” and “Glove 2”, which consisted of colour images and depth images, were recorded with a Kinect for this study and then used to evaluate the proposed algorithm (elaborated in Section 3.1, Section 3.2, Section 3.3). The total frame number of all image sequences was 6512.

**Table 2:** Challenges of the benchmark sequences.

Sequences		Major Challenge
Standard	Coke	Fast motion, Occlusion, Background Clutters
	Couple	Fast Motion, Deformation, Background Clutters
	Liquid	Occlusion, Fast Motion, Background Clutters
	Suv	Occlusion, In-Plane Rotation, Out-of-View
	Tiger1	Illumination Variation, Fast Motion, Occlusion
	Tiger2	Illumination Variation, Fast Motion, Occlusion
	Fish	Out-of-View
Ours	Note	Fast Motion, Occlusion
	Book	Random Motion
	Glove 1	Random Motion, Occlusion
	Glove 2	Random Motion, Leave Field of View

Table 3 shows the quantitative evaluation results of the benchmark test. In the test of the improved CT algorithm, the sampling radius was set to γ = 50, the dimensionality of compressed feature vectors was set to *n* = 50 and the learning parameter was set to λ = 0.85.

**Table 3:** Success rate (SR) and centre location error (CLE) of the improved CT.

Sequences		Improved CT		Original CT		MILTrack		Number of Frames
		SR	CLE	SR	CLE	SR	CLE	
Standard	Coke	19.2	31.2	15.8	36.1	9.2	28.8	291
	Couple	83.6	8.6	68.6	35.4	65.7	35.9	140
	Liquor	40.1	141.9	28.1	168.9	21.0	131.8	1741
	Suv	58.5	39.1	17.9	79.2	46.2	59.8	945
	Tiger1	49.3	23.6	46.8	28.0	8.5	104.6	354
	Tiger2	51.7	24.5	45.2	28.6	45.7	28.3	365
	Fish	9.5	37.1	3.8	38.8	81.3	10.2	476
Frames per Second		61.4		63.1		32.5		

**Figure 9:**
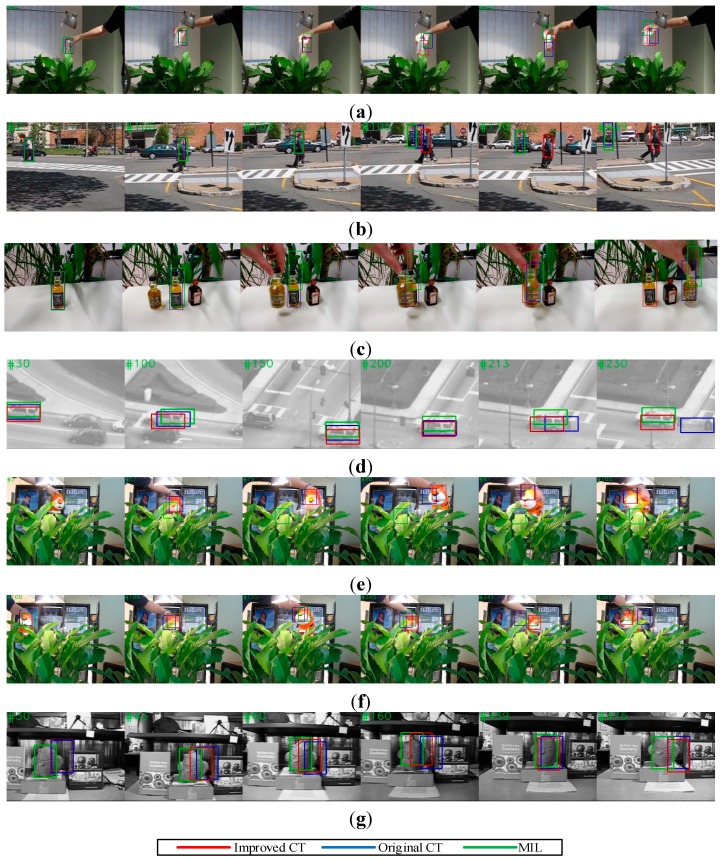
Tracking result on classical image sequences. (**a**) Tracking results of “Coke”; (**b**) Tracking results of “Couple”; (**c**) Tracking results of “Liquor”; (**d**) Tracking results of “Suv”; (**e**) Tracking results of “Tiger1”; (**f**) Tracking results of “Tiger2”; and (**g**) Tracking results of “Fish”.

The results indicated that the robustness to fast motion, occlusion and background clutter of the improved CT algorithm was reinforced by the motion estimation mechanism, with only a slight increase in the computational complexity of the improved CT algorithm, which did not influence its real-time performance. As shown in Figure 9a, the bounding box of CT and MIL obviously drifted when the Coke can in the image moved erratically at #200 and #225, whereas the improved CT tracker was able to track the target precisely.

As shown in Figure 9f, the CT tracker completely lost the target when occlusion occurred frequently at #341 and #362, whereas the improved CT tracker successfully detected the target when it reappeared. As shown in Figure 9b, the CT tracker and MIL tracker both missed the couple in the image when a car with similar Haar-like features appeared, but the improved CT tracker avoided this failure by predicting the motion trend of the target. Although the improved CT algorithm had a higher success rate in the benchmark test, it made little contribution to the performance improvement when illumination variation or full occlusion occurred, as shown in Figure 9c,g. Furthermore, as shown in Figure 9e, if the target moved randomly or too rapidly, motion estimation based on a Kalman filter may not have improved and may even have diminished the effectiveness of the CT algorithm, as the Kalman filter only works well for Gaussian linear systems. This result demonstrated that a backup detection mechanism to supervise the improved CT tracker is essential.

**Figure 10:**
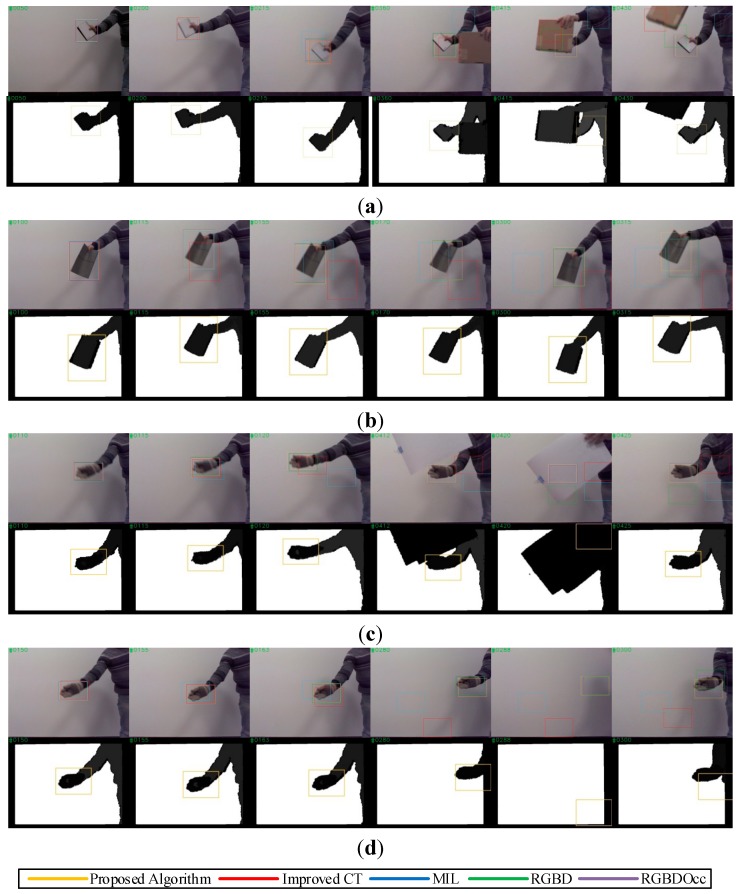
Tracking results of our image sequences in real scenarios. (**a**) Tracking results of “Note”; (**b**) Tracking results of “Book”; (**c**) Tracking results of “Glove1”; and (**d**) Tracking results of “Glove2”.

Table 4 shows the quantitative evaluation results of the actual test. In the test of the proposed algorithm, the sampling radius was set to γ = 50, the dimensionality of compressed feature vectors was set to *n* = 50 and the learning parameter was set to λ = 0.85. The results indicated that the effectiveness and robustness of the proposed algorithm were further improved with the multi-feature fusion mechanism and the feedback strategy. Although the tracking precision and robustness of RGBDOcc were better, it seemed that the proposed algorithm is more suitable to robotic applications in consideration of the real-time performance. As shown in Figure 10a, the MIL tracker first missed the target when the target moved at a fast speed for a long time at #215, and the improved CT tracker later failed after full occlusion. However, the tracker using the proposed algorithm, which stopped updating the classifier when full occlusion occurred, was subsequently able to detect the target and reset the depth tracker successfully at #430. As shown in Figure 10b,c, the improved CT tracker and the MIL tracker failed in tracking targets that moved randomly or swerved erratically, but the tracker based on the proposed algorithm succeeded by extending the search region and adjusting parameters of trackers.

**Table 4:** Success rate (SR) and centre location error (CLE) of the proposed algorithm.

Sequences		Proposed Algorithm		Improved CT		MILTrack		RGBD Baseline		RGBDOcc		Number of Frames
		SR	CLE	SR	CLE	SR	CLE	SR	CLE	SR	CLE	
Ours	Note	87.2	18.3	62.1	22.6	57.2	27.2	72.1	21.7	91.1	13.9	600
	Book	88.5	9.7	36.6	55.3	46.3	47.9	76.8	17.9	86.3	9.1	600
	Glove1	85.5	12.3	43.5	29.1	40.9	63.6	69.3	41.8	88.3	8.3	600
	Glove2	79.2	17.5	47.2	23.5	41.6	42.7	59.4	49.3	75.1	16.2	400
Frames per Second		39.7		57.3		31.7		<1.0		<1.0		

As shown in Figure 10d, the tracker using the proposed algorithm was able to handle the scenario in which the target left the field of view and reappeared later by temporarily stopping online learning. The test results verified the effectiveness and robustness of the proposed algorithm and the feasibility of designing a robust tracking system with compound sensors, such as the Kinect.

**Figure 11:**
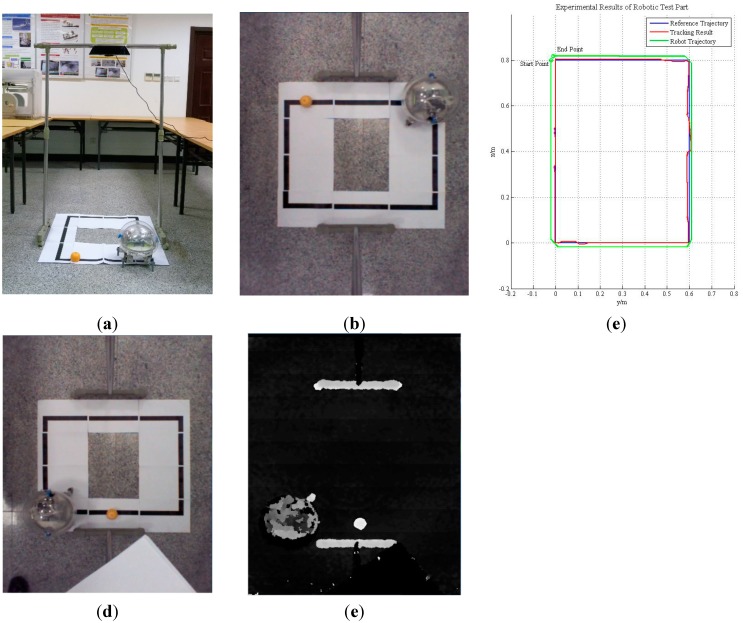
Robotic tracking experiment with our amphibious spherical robot. (**a**) Experimental scenario; (**b**) Perspective of Kinect; (**c**) Experimental Results; (**d**) Colour Image before Occlusion; and (**e**) Depth Image before Occlusion.

In the robotic test, a prototype of the robotic tracking system was built for algorithm evaluation. As it is too large to be installed in our robot, the Kinect was fixed on a frame, and monitored the motion of our spherical robot from overhead, as shown in Figure 11a, while a small orange hemisphere that moved along a black box measuring 0.8 m × 0.6 m acted as a guiding object. As shown in Figure 8 and Figure 11b, the proposed algorithm running on the computer tracked the guiding object, calculated its trajectory and controlled the motion of our spherical robot via the wireless local area network. When the guiding object arrived at the position (0.6, 0.4), it was fully occluded by a book for several seconds. Figure 11c shows the tracking results (red lines) and the trajectory of the robot (green lines). There was only slight deviation of less than 5 cm or 3 pixels between the tracking result and the designated trajectory, and the controller module of the robot was set to omit small deviations or noise. As a result, except for drift occurring at bends in the designated trajectory, the robot was able to follow the motion of the guiding object almost exactly with the guidance of the tracking system. Even the target was fully occluded, the tracking system detected the target after slight drift when the target reappeared.

## 5. Conclusions

In this paper, a real-time visual tracking algorithm was proposed and implemented for our amphibious spherical robot. To meet the requirements of robots in robustness, adaptability and effectiveness of tracking systems, Microsoft Kinect was adopted to capture colour and depth images. An improved CT tracker with a Kalman prediction mechanism was deployed to process colour images from the Kinect, and a VR-V tracker was implemented to process depth images from the Kinect. The update rate of the CT tracker was adjusted using a feedback strategy, which partly solved the problem of its ineffectiveness in situations of full occlusion, irregular target motion and illumination variation. The experimental results of virtual, actual and robotic tests verified the effectiveness, efficiency and robustness of the proposed algorithm.

This paper presents only a preliminary prototype of the robotic tracking system. Kinect cannot be installed in our spherical robot due to size limitations. Also, Kinect is not able to provide depth images of high quality in outdoor and underwater environment because it acquire depth information with an infrared speckle dot pattern projector and an infrared camera [33]. Furthermore, the mechanisms of parameter adjustment and adaptive classifier updating in the feedback strategy were experience-based and primitive in some measures. Future work will focus on realisation of a small Kinect-like compound image sensor which consists of a colour camera and a laser-based depth camera for applications in amphibious environments [43].
